# Demographic, socio-economic, obstetric, and behavioral factors associated with small-and large-for-gestational-age from a prospective, population-based pregnancy cohort in rural Nepal: a secondary data analysis

**DOI:** 10.1186/s12884-022-04974-8

**Published:** 2022-08-19

**Authors:** Elizabeth A. Hazel, Diwakar Mohan, Scott Zeger, Luke C. Mullany, James M. Tielsch, Subarna K. Khatry, Seema Subedi, Steven C. LeClerq, Robert E. Black, Joanne Katz

**Affiliations:** 1 Department of International Health, Johns Hopkins Bloomberg School of Public Health, 615 N. Wolfe St. Room W5504, Baltimore, MD 21205 USA; 2 George Washington University Milken Institute School of Public Health, Washington, DC USA; 3 Nepal Nutrition Intervention Project-Sarlahi, Sarlahi, Nepal

**Keywords:** Small-for-gestational age, Large-for-gestational age, Cohort study, Nepal

## Abstract

**Background:**

In South Asia, a third of babies are born small-for-gestational age (SGA). The risk factors are well described in the literature, but many studies are in high-and-middle income countries or measure SGA on facility births only. There are fewer studies that describe the prevalence of risk factors for large-for-gestational age (LGA) in low-income countries. We aim to describe the factors associated with SGA and LGA in a population-based cohort of pregnant women in rural Nepal.

**Methods:**

This is a secondary data analysis of community-based trial on neonatal oil massage (22,545 women contributing 39,479 pregnancies). Demographic, socio-economic status (SES), medical/obstetric history, and timing of last menstruation were collected at enrollment. Vital signs, illness symptoms, and antenatal care (ANC) attendance were collected throughout the pregnancy and neonatal weight was measured for live births. We conducted multivariate analysis using multinomial, multilevel logistic regression, reporting the odds ratio (OR) with 95% confidence intervals (CIs). Outcomes were SGA, LGA compared to appropriate-for-gestational age (AGA) and were multiply imputed using birthweight recalibrated to time at delivery.

**Results:**

SGA was associated with nulligravida (OR: 2.12 95% CI: 1.93–2.34), gravida/nulliparous (OR: 1.86, 95% CI: 1.26–2.74), interpregnancy intervals less than 18 months (OR: 1.16, 95% CI: 1.07–1.27), and poor appetite/vomiting in the second trimester, (OR: 1.27, 95% CI: 1.19–1.35). Greater wealth (OR: 0.78, 95% CI: 0.69–0.88), swelling of hands/face in the third trimester (OR: 0.81, 95% CI: 0.69–0.94) parity greater than five (OR: 0.77, 95% CI: 0.65–0.92), male fetal sex (OR: 0.91, 95% CI: 0.86–0.98), and increased weight gain (OR: 0.93 per weight kilogram difference between 2^nd^ and 3^rd^ trimester, 95% CI: 0.92–0.95) were protective for SGA.

Four or more ANC visits (OR: 0.53, 95% CI: 0.41–0.68) and respiratory symptoms in the third trimester (OR: 0.67, 95% CI: 0.54–0.84) were negatively associated with LGA, and maternal age < 18 years (OR: 1.39, 95% CI: 1.03–1.87) and respiratory symptoms in the second trimester (OR: 1.27, 95% CI: 1.07–1.51) were positively associated with LGA.

**Conclusions:**

Our findings are in line with known risk factors for SGA. Because the prevalence and mortality risk of LGA babies is low in this population, it is likely LGA status does not indicate underlaying illness. Improved and equitable access to high quality antenatal care, monitoring for appropriate gestational weight gain and increased monitoring of women with high-risk pregnancies may reduce prevalence and improve outcomes of SGA babies.

**Trial Registration:**

The study used in this secondary data analysis was registered at Clinicaltrials.gov NCT01177111.

**Supplementary Information:**

The online version contains supplementary material available at 10.1186/s12884-022-04974-8.

## Background

In 2012, an estimated 23.3 million babies were born small-for-gestational age in low-and-middle income countries (LMICs) [1]. Compared to appropriate-for-gestational age babies, SGA babies have an 83% higher risk of dying in the first month of life and are at increased risk of faltering physical and neurological development [2, 3] Small-for-gestational age (SGA) is defined as a birthweight-for-gestational age less than the 10^th^ percentile of a sex-specific standard infant population. Globally, South Asia has the highest prevalence, a third of babies are born SGA (34%) and SGA accounts for a quarter (24%) of all neonatal deaths [1].

There have been many studies on the risk factors for small newborns across high-and low- income and prevalence settings, looking at both SGA and low birthweight (birthweight < 2500 g irrespective of gestational age) as outcomes but many are hospital-based [4–7] or based on maternal recall of birth weight from household surveys [8, 9]. Babies born at home may be at higher risk of SGA and maternal report of birthweight from household surveys may be impacted by recall bias and have high levels of missingness [10].

Risk factors previously identified include socioeconomic (i.e., education, marital status), reproductive history (i.e., adolescent birth, parity), access to health care (i.e., tetanus toxoid vaccination, antenatal care), behavioral factors (i.e., tobacco and caffeine use), and maternal health factors (i.e., hypertension, eclampsia/preeclampsia or disease such as HIV infection or periodontal disease) [4, 11–14]. A recent analysis of 81 LMICs found vitamin D deficiency, low gestation weight gain, hypertension, primiparity between 18–35 years of age, short height, and air pollution to be the leading population attributable risk factors for SGA [15]. However many of the studies evaluating risk factors focus on low birthweight as an outcome or are not population-based. Additionally, large-for-gestational age (LGA) babies with a birthweight > 90^th^ percentile of a standard population have associated health risks but less is known about the risk factors or prevalence of disease in LGA babies born in LMICs [16, 17].

To estimate SGA and LGA, an accurate measure of gestational age and birthweight at delivery is required, along with infant sex. Birthweight measures of babies born at home are critical to estimate population-level SGA, but it may take several days before a health worker can measure weights of infants born at home, often at the nadir of the infant’s weight loss. Typically, babies may lose up to 10% of their body weight in the early neonatal period, primarily due to loss of water weight, as they physiologically adjust to life outside the womb and this weight loss is not indicative of poor health [18]. Using birthweights imputed to the time of delivery reduces over-estimation of SGA [19].

In this study, we aim to determine risk factors for SGA and LGA including maternal, demographic, socioeconomic, health access, and pregnancy level factors in a South Asian setting with high prevalence of SGA. This study contributes to the body of knowledge on risks for SGA and LGA in LMICs, using data collected prospectively throughout pregnancy, including both facility and home births, and is one of the first to measure these associations using birthweights imputed to the time of delivery.

## Methods

This is a secondary data analysis of a community-based, randomized controlled trial in the Sarlahi district in southern Nepal (Clinicaltrials.gov NCT01177111). In this setting, approximately a fifth of all neonatal deaths are due to infection.^1^ Traditionally, babies are massaged with mustard oil – a substance that may reduce the structural integrity of the skin barrier, resulting in increased infections. It was hypothesized that improving skin barrier function through oil massage with a less irritating substance could make babies less susceptible to infections, especially those born preterm and/or SGA. The Nepal Oil Massage Study (NOMS) aimed to determine whether neonatal massage using sunflower oil, instead of traditional mustard oil reduced neonatal morbidity and mortality [20].

### Data collection

**Fig. 1:**
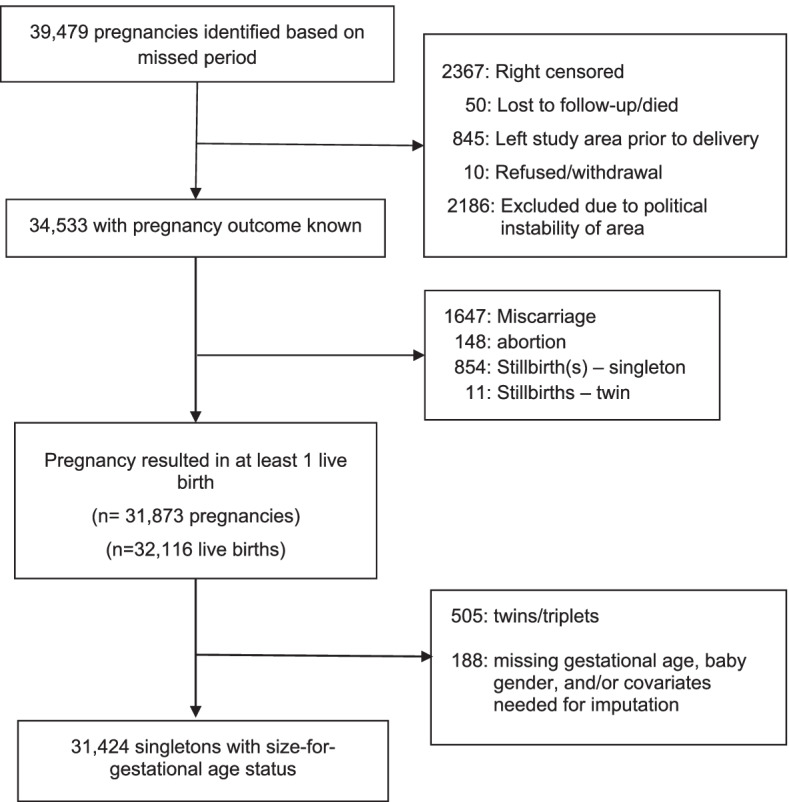
Flow diagram of pregnancies, losses, outcomes, follow up, Nepal Oil Massage Study

Geographic areas (340 clusters) were randomized to control (mustard oil, *n* = 171 clusters) or treatment (sunflower oil, *n* = 169 clusters) prior to the start of the study. Pregnant women were identified in the community by field workers at the Nepal Nutritional Intervention Project-Sarlahi site. All pregnant women (regardless of age) living in the study area were eligible. Those who consented verbally were visited at home approximately every five weeks to collect data on last menstrual period (LMP). If the woman had not had a menstrual period in the previous month, she was offered a pregnancy test and if pregnant, she was enrolled in the study. Median gestational age at enrollment was 14.1 weeks and some women contributed multiple pregnancies to the study. Enrollment occurred from November 2010 through January 2017, with vital data on neonates collected through July 2017. From the 340 clusters, 39,479 pregnancies were identified, 34,533 pregnancies with a known outcome and 32,116 live births (Fig. 1).

At enrollment, data were collected on household demographic factors, socioeconomic status and women’s age, height in centimeters (cm), alcohol and tobacco use, and reproductive history. The field teams visited the study participants monthly during their pregnancy to administer a questionnaire on any symptoms they experienced and to measure blood pressure, weight, pulse, and temperature. Mothers were visited shortly after the delivery for additional data collection on the place of delivery, number of antenatal care visits, exact date and time of delivery, infant sex, singleton or multiple status, and measurement of infant weight. Morbidity and mortality data were collected through the neonatal period (28 days).

Weight was measured in grams using a Tanita digital infant weight scale with 10 g precision. Weight for both home and facility births were measured by trained study staff with sufficient inter-and interobserver reliability. Scales were recalibrated daily and evaluated throughout the study for drift. Weight was measured three times and recorded the median. The team collected weight only on liveborn babies who survived to the time of the first postnatal visit. Median time of weight measurement was 15.2 h: 12.3 h for babies born at home and 19.8 h for babies born at the facility.

### Outcome variable definition and imputation

Gestational age was calculated using the difference between the date of LMP collected at enrollment and the reported date and time of delivery. Since weights were measured outside the first 24 h after delivery for approximately a third of the infants (33.7%), we conducted an imputation that [1] pulls the weights measured during the early neonatal period back to the time of delivery; [2] imputes a birthweight for babies missing weight measurement (12% of the sample). Additional details on the imputation methods are found here [19].

SGA status was defined using Intergrowth-21^st^ population standards stratified by infant sex that were extrapolated for 42–44 and 22–24 weeks GA.^2^ [21] Our primary outcomes were SGA (< 10 percentile (pc) compared to the standard population) and LGA (> 90pc). Babies missing gender (0.3%) required to determine SGA status were excluded. No babies were missing gestational age.

### Covariate definitions

Socioeconomic status of the mother was defined by reported, education of the mother was categorized by years, caste/religion, and wealth was defined by principle components analysis of household assets ownership by quintile [22]. Caste/religion (*Brahmin* /*Chhetri*, *Vaishya*, *Shudra*, Muslim and others) was defined using the Nepal caste system [23]. We defined alcohol or tobacco fetal exposure as any reported use during the pregnancy (which was asked monthly). Babies were considered protected from tetanus toxoid if the mother reported at least one vaccine dose in the two years before the pregnancy at the time of enrollment or during the pregnancy. Since antenatal care (ANC) was relatively low, we categorized ANC as no visits as the highest risk, followed by at least one visit of ANC, 2–3 and four or more. Birth in a health post, clinic or hospital was defined as a facility delivery and delivery at home, at the parents’ house (*maiti*) or enroute to the facility or outdoors were defined as non-facility deliveries.

Reported symptoms during the pregnancy were grouped by type of symptom and trimester. Any reported coughing, difficulty breathing, wheezing or shortness of breath was defined as respiratory illness. Swelling symptoms were defined as swelling of the hands and face only. Foot/leg swelling was excluded since it is common during pregnancy and not indicative of underlying disease. Symptoms of sexually transmitted infection were reported as painful urination or foul-smelling vaginal discharge. Symptoms of upper gastrointestinal illness were poor appetite, vomiting and nausea and lower gastrointestinal illness were watery stool or presence of blood/mucus in the stool. Vaginal bleeding included any bleeding or spotting during the pregnancy.

High systolic blood pressure was ≥ 140 and high diastolic pressure was ≥ 90 mm of mercury (mmHG) at any time during the trimester, as measured by the study team. Maternal weight was measured, and symptom data were collected at every pregnancy visit. We excluded the first trimester symptoms and vital signs because more than half of the women are missing first trimester data (58.6%) due to a relatively small number of women recruited in their first trimester. A fifth of women (19.9%) were missing second trimester data and a tenth were missing third trimester data. We analyzed the mean weight change from 2^nd^ to 3^rd^ trimester in kilograms (kg). Symptom and vital data were categorized by presences or absence during the second and third trimester.

### Analysis

We conducted bivariate and multivariate analysis using multinomial, multilevel logistic regression, clustered by women to account for women who contributed more than one pregnancy to the study. The outcomes were SGA (< 10pc), LGA (> 90pc) compared to AGA (10-90pc) and were multiply imputed using birthweight recalibrated to time 0, at delivery. Covariates in the adjusted model were selected and categorized both empirically and based on knowledge of risk factors for SGA. We report the odd ratio with 95% confidence intervals to determine whether covariates are statistically associated with SGA and may be considered a risk factor. We used Stata 16.1 for all analysis [24]. We excluded twins/triplets from this analysis since it is well-documented they are at high risk of SGA and the etiology is different from singletons.

## Results

### Descriptive

Of the 32,116 live births, 505 twins/triplets and babies with missing gestational age, gender and/or covariates needed for the imputation were excluded. There were 31,424 singleton babies (98% of the live births) included in the analysis (Fig. 1). Over half of the babies were born to women with no formal schooling (67%), 15% to women with short stature (< 145 cm) and 16% of the mothers were less than 18 years old. (Tables 1 & 2). About a third of the babies were their mother’s first pregnancy and among those with a previous pregnancy, 6% experienced a prior stillbirth, 16% had a miscarriage and 16% had a live born child that was now deceased. A third of the women had an interpregnancy interval of less than 18 months and 64% had one to four previous live-or-stillbirths. Missing covariate data on obstetric history was low (< 2%) (Tables 1& 2).

**Table 1:** Socio-economic Characteristics, fixed for mother: total and by size-for-gestational age category

	Total infants (%)	AGA (90-10pc) % (95% CI)	SGA (< 10pc) % (95% CI)	LGA (> 90pc) % (95% CI)
	*n* = 31,424			
**Mother's religion/ caste**				
*Brahmin & Chhetri*	3.0	59.9 (56.4,63.4)	35.8 (32.4,39.2)	4.3 (2.7,5.8)
*Vaishya*	72.1	51.3 (50.4,52.1)	44.4 (43.6,45.3)	4.3 (4.1,4.6)
*Shudra*	15.5	45.4 (43.7,47.1)	50.0 (48.4,51.6)	4.6 (4.0,5.2)
*Muslim and others*	9.3	53.8 (51.7,55.9)	38.8 (36.7,40.9)	7.4 (6.4,8.4)
*Missing*	0.1	-	-	-
**Mother's Education**				
*No schooling*	67.3	49.1 (48.3,49.9)	45.8 (45.0,46.6)	5.1 (4.8,5.4)
*1 to 5years*	8.6	50.2 (48.0,52.3)	45.6 (43.5,47.7)	4.2 (3.4,5.1)
*5* + *years*	24.2	55.9 (54.6,57.3)	40.5 (39.2,41.7)	3.6 (3.0,4.2)
**Wealth Quintile**				
*Poorest*	20.3	45.9 (44.5,47.2)	48.9 (47.6,50.2)	5.2 (4.7,5.8)
*Poorer*	20.0	49.4 (47.9,50.9)	45.9 (44.4,47.4)	4.7 (4.1,5.2)
*Middle*	19.9	50.6 (49.3,51.9)	44.4 (43.1,45.8)	4.9 (4.3,5.5)
*Richer*	19.8	52.1 (50.5,53.7)	43.7 (42.2,45.2)	4.2 (3.7,4.8)
*Least poor*	20.0	56.4 (54.8,58.0)	39.4 (38.0,40.9)	4.2 (3.6,4.7)
*Missing*	0.1	-	-	-
**Mother's height (centimeters)**				
< 145	14.7	41.4 (39.8,42.9)	54.8 (53.2,56.3)	3.9 (3.3,4.4)
145–149	30.0	47.3 (46.2,48.4)	48.2 (47.1,49.3)	4.5 (4.1,5)
≥ 150	55.1	55.3 (54.5,56.2)	39.8 (38.9,40.6)	4.9 (4.6,5.3)
Missing	0.2	-	-	-

*AGA* Appropriate for gestational age, *SGA* Small for gestational age, *LGA* Large for gestational age, *pc* Percentile, *CI* Confidence interval

**Table 2:** Demographic, Obstetric, and Behavoiral Characteristics, and symptoms experienced during pregnancy, varying by pregnancy: total and by size-for-gestational age category

	Total infants (%)	AGA (90-10pc) % (95% CI)	SGA (< 10pc) % (95% CI)	LGA (> 90pc) % (95% CI)
	*n* = 31,424			
**Age at last menstrual period**				
18–35	82.2	52.4 (51.7,53.1)	42.9 (42.2,43.6)	4.7 (4.4,4.9)
Less than 18	15.6	42.3 (40.5,44.1)	54.1 (52.3,55.9)	3.6 (3.1,4.2)
More than 35	2.2	54.5 (50.6,58.3)	34.6 (30.5,38.7)	10.9 (8.4,13.5)
**Parity (still- and livebirths)**				
Parity 1–4	63.7	54.4 (53.6,55.2)	40.4 (39.6,41.2)	5.2 (4.9,5.5)
More than 4	4.3	56.4 (53.5,59.2)	34.4 (31.8,36.9)	9.2 (7.5,11)
Prior pregnancy, nulliparous	2.5	44.4 (40.5,48.3)	51.9 (47.9,55.8)	3.7 (2.0,5.4)
No prior pregnancy	29.0	42.7 (41.5,44.0)	54.5 (53.3,55.8)	2.8 (2.4,3.1)
Missing	0.5	-	-	-
**Any Prior Livebirth Died**				
Prior livebirth, no death	54.9	54.7 (53.8,55.6)	40.0 (39.2,40.9)	5.3 (4.9,5.6)
Prior livebirth death	11.3	53.9 (52.2,55.7)	39.7 (38,41.5)	6.3 (5.5,7.2)
Prior pregnancy, no livebirth	3.4	47.4 (43.9,50.9)	49.1 (45.4,52.9)	3.5 (2.2,4.7)
No prior pregnancy	29.0	42.7 (41.5,44.0)	54.5 (53.3,55.8)	2.8 (2.4,3.1)
Missing	1.5	-	-	-
**Any Prior Stillbirth**				
Prior pregnancy, no stillbirth	66.8	54.2 (53.3,55.0)	40.4 (39.6,41.2)	5.4 (5.1,5.8)
Prior stillbirth	4.3	54.6 (51.5,57.8)	40.3 (37.3,43.3)	5.1 (3.8,6.3)
No prior pregnancy	29.0	42.7 (41.5,44.0)	54.5 (53.3,55.8)	2.8 (2.4,3.1)
Missing	0	-	-	-
**Any Prior Miscarriage**				
Prior pregnancy, no miscarriage	59.7	54.5 (53.6,55.3)	39.9 (39.0,40.7)	5.7 (5.3,6.0)
Prior miscarriage	11.4	52.6 (50.9,54.4)	43.1 (41.4,44.8)	4.2 (3.5,4.9)
No prior pregnancy	29.0	42.7 (41.5,44.0)	54.5 (53.3,55.8)	2.8 (2.4,3.1)
Missing	0	-	-	-
**Interpregnancy interval (months)**				
18–36	24.8	56.2 (54.9,57.5)	38.4 (37.2,39.7)	5.4 (4.8,5.9)
< 18	36.0	51.7 (50.7,52.8)	42.9 (41.8,43.9)	5.4 (5.0,5.8)
> 36	10.2	57.9 (55.3,60.4)	36.6 (34.1,39.1)	5.5 (4.7,6.4)
No prior pregnancy	29.0	42.7 (41.5,44.0)	54.5 (53.3,55.8)	2.8 (2.4,3.1)
Missing	0	-	-	-
**Tobacco use during pregnancy**				
No	98.9	50.9 (50.3,51.6)	44.5 (43.9,45.1)	4.6 (4.4,4.8)
Yes	1.1	44.4 (38.9,50.0)	45.8 (40.0,51.6)	9.7 (6.5,13)
Missing	0	-	-	-
**Alcohol use during pregnancy**				
No	99.7	50.9 (50.2,51.5)	44.5 (43.9,45.1)	4.6 (4.4,4.9)
Yes	0.3	54 (43.5,64.6)	36.6 (26.4,46.8)	9.4 (3.2,15.6)
Missing	0	-	-	-
**At least one dose of tetanus toxoid vaccine taken in previous two years**				
No	15.8	49.5 (47.9,51.1)	44.1 (42.5,45.7)	6.4 (5.6,7.2)
Yes	84.2	51.1 (50.4,51.8)	44.6 (43.9,45.3)	4.3 (4.1,4.6)
Missing	0	-	-	-
**Number of antenatal care visits**				
No visit	17.4	49.0 (47.5,50.5)	44.2 (42.7,45.6)	6.8 (6.1,7.5)
1 visit	13.1	50.5 (48.9,52.2)	43.6 (42.0,45.2)	5.9 (5.1,6.6)
2–3 visit	30.7	51.4 (50.2,52.5)	43.9 (42.8,45)	4.8 (4.3,5.2)
4 or more	28.1	53.3 (52.0,54.6)	43.9 (42.6,45.2)	2.8 (2.4,3.2)
Missing	10.7	-	-	-
**Place of Delivery**				
Home/maiti	49.8	50.8 (49.9,51.7)	43.9 (43,44.8)	5.3 (5.0,5.7)
Health post/clinic/hospital	37.8	52.1 (50.9,53.3)	44.0 (42.9,45.2)	3.9 (3.5,4.2)
On way to facility/outdoor	1.9	50.8 (46.0,55.6)	43.9 (39.6,48.3)	5.2 (3.2,7.3)
Missing	10.6	-	-	-
**Infant gender**				
Male	51.8	51.8 (50.9,52.7)	43.3 (42.5,44.2)	4.9 (4.5,5.2)
Female	48.2	49.8 (48.9,50.7)	45.7 (44.8,46.7)	4.4 (4.1,4.8)
**Sexually transmitted infection symptoms, 2**^**nd**^** trimester**				
No	65.3	51.2 (50.5,51.9)	43.6 (42.9,44.3)	5.2 (4.8,5.5)
Yes	14.4	50.5 (48.8,52.1)	44.4 (42.5,46.2)	5.2 (4.5,5.9)
Missing	20.1	-	-	-
**Sexually transmitted infection symptoms, 3**^**rd**^** trimester**				
No	81.6	51.8 (51.2,52.5)	44.8 (44.1,45.5)	3.4 (3.1,3.6)
Yes	9.3	50.3 (48.3,52.3)	47.0 (45.0,49)	2.7 (2.1,3.3)
Missing	9.1	-	-	-
**Respiratory illness, 2**^**nd**^** trimester**				
No	56.3	51.4 (50.6,52.1)	43.6 (42.8,44.3)	5.1 (4.7,5.4)
Yes	23.4	50.4 (49.1,51.7)	44.2 (42.8,45.5)	5.4 (4.9,6.0)
Missing	20.3	-	-	-
**Respiratory illness, 3**^**rd**^** trimester**				
No	71.9	52.2 (51.4,52.9)	44.3 (43.6,45.1)	3.5 (3.3,3.8)
Yes	19.0	49.9 (48.5,51.3)	47.7 (46.4,49.0)	2.4 (2.0,2.8)
Missing	9.1	-	-	-
**Gastrointestinal illness, 2**^**nd**^** trimester**				
No	71.4	51 (50.3,51.7)	43.8 (43.1,44.5)	5.2 (4.9,5.5)
Yes	8.4	52 (49.8,54.2)	43.1 (41.0,45.3)	4.9 (4.0,5.7)
Missing	20.3	-	-	-
**Gastrointestinal illness, 3**^**rd**^** trimester**				
No	82.3	51.8 (51.0,52.5)	44.9 (44.1,45.6)	3.3 (3.1,3.6)
Yes	8.6	50.7 (48.4,52.9)	46.6 (44.2,48.9)	2.7 (2.1,3.4)
Missing	9.1	-	-	-
**Poor appetite, vomiting, 2**^**nd**^** trimester**				
No	41.2	53.1 (52.1,54.1)	40.9 (39.9,42.0)	6.0 (5.5,6.4)
Yes	38.6	48.9 (48.0,49.8)	46.7 (45.8,47.7)	4.4 (4.0,4.7)
Missing	20.2	-	-	-
**Poor appetite, vomiting, 3**^**rd**^** trimester**				
No	70.7	52.5 (51.7,53.4)	44.0 (43.2,44.8)	3.5 (3.2,3.8)
Yes	20.2	48.8 (47.5,50.1)	48.7 (47.4,50.0)	2.5 (2.1,3.0)
Missing	9.1	-	-	-
**Vaginal bleeding, 2**^**nd**^** trimester**				
No	78.6	51.0 (50.4,51.7)	43.8 (43.1,44.5)	5.2 (4.9,5.5)
Yes	1.2	55.6 (49.6,61.6)	39.9 (33.9,45.9)	4.5 (2.3,6.8)
Missing	20.3	-	-	-
**Vaginal bleeding, 3**^**rd**^** trimester**				
No	90.4	51.7 (51,52.4)	45.0 (44.3,45.7)	3.3 (3.1,3.5)
Yes	1.0	47.4 (39.3,55.6)	49.8 (41.9,57.8)	2.7 (0.0,5.7)
Missing	9.1	-	-	-
**Swelling hand/face, 2**^**nd**^** trimester**				
No	78.0	51.1 (50.4,51.7)	43.9 (43.2,44.5)	5.1 (4.8,5.4)
Yes	1.8	52.1 (47.9,56.4)	38.0 (33.8,42.3)	9.8 (7.1,12.5)
Missing	20.2	-	-	-
**Swelling hands/face, 3**^**rd**^** trimester**				
No	87.4	51.5 (50.8,52.2)	45.2 (44.6,45.9)	3.3 (3,3.5)
Yes	3.6	56.0 (52.9,59.1)	40.0 (36.9,43)	4.0 (2.8,5.3)
Missing	9.1	-	-	-
**High diastolic, 2**^**nd**^** trimester**				
No	78.3	51.1 (50.5,51.8)	43.8 (43.1,44.4)	5.1 (4.8,5.4)
Yes	1.5	49.1 (44.1,54.1)	41.5 (36.2,46.7)	9.4 (6.7,12.2)
Missing	20.2	-	-	-
**High diastolic, 3**^**rd**^** trimester**				
No	88.1	51.8 (51.1,52.5)	44.9 (44.3,45.6)	3.3 (3.0,3.5)
Yes	2.8	47.7 (43.3,52.1)	47.9 (43.5,52.3)	4.4 (2.9,5.9)
Missing	9.1	-	-	-
**High systolic 2**^**nd**^** trimester**				
No	79.3	51.1 (50.4,51.7)	43.7 (43.1,44.4)	5.2 (4.9,5.5)
Yes	0.5	49.5 (40.9,58.0)	44.1 (35.3,52.9)	6.4 (2.3,10.5)
Missing	20.2	-	-	-
**High systolic 3**^**rd**^** trimester**				
No	90.2	51.7 (51.0,52.4)	45 (44.3,45.6)	3.3 (3.1,3.5)
Yes	0.7	47.3 (39.1,55.5)	49.1 (40.8,57.4)	3.6 (0.9,6.4)
Missing	9.1	-	-	-
**Mean weight change in kilograms (kg) from 2**^**nd**^** to 3**^**rd**^** trimester**	3.5 kg	3.72 kg (3.7, 3.8)	3.3 kg (3.3, 3.4)	3.3 kg (3.1–3.5)

*AGA* Appropriate for gestational age, *SGA* Small for gestational age, *LGA* Large for gestational age, *pc* Percentile, *CI* Confidence interval

Half of the babies were born at home, and 28% had four or more antenatal care visits. Ten percent of the babies had missing place of delivery and/or antenatal care data. During pregnancy, 84% of the mothers received at least one dose of tetanus toxoid vaccine and very few used alcohol or tobacco (< 2%). The most common symptom reported in either the second or third trimester was poor appetite/vomiting (39% in the second; 20% in the third) and the rarest was vaginal bleeding (1.2% in the second and 1.0% in the third). High diastolic or systolic blood pressure was rare, less than 3% measured in either the second or third trimester. On average, women gained 3.5 kg from the second to third trimester (Table 2).

### Risk factors for small-for-gestational age

SGA was associated with socio-economic status of the mother. Women with five or more years of formal schooling (OR: 0.75, 95% CI 0.69–0.82) and from wealthier households has reduced odds of having an SGA baby (OR: 0.78, 95% CI 0.69–0.88 for the wealthiest households compared to the poorest). Lower caste was associated with SGA as well (Table 3). Maternal height 145–149 cm (cm) (OR: 0.75, 95% CI 0.68–0.83) and greater than 150 cm (OR: 0.52, 95% CI: 0.47–0.57)was protective of SGA compared to women of short stature (< 145 cm). Advanced (> 35 years) or young (< 18 years) maternal age had no statistically significant association with SGA.

**Table 3:** Adjusted odds ratio of small-for-gestational age and large for gestational age by covariate

	**Small-for gestational age**			**Large-for-gestational age**		
	**Odds ratio**	**95% CI—LL**	**95% CI—UL**	**Odds ratio**	**95% CI—LL**	**95% CI—UL**
**Mother's religion/ caste**						
Brahmin & Chhetri	*ref*	*ref*	*ref*	*ref*	*ref*	*ref*
Vaishya	1.37	1.13	1.66	1.01	0.58	1.77
Shudra	1.48	1.21	1.81	1.01	0.56	1.82
Muslim and others	1.04	0.84	1.28	1.59	0.86	2.94
**Mother's Education**						
No schooling	*ref*	*ref*	*ref*	*ref*	*ref*	*ref*
1–5 years	0.97	0.86	1.10	1.06	0.79	1.44
> 5 years	0.77	0.71	0.84	0.84	0.65	1.10
**Women age at last menstrual period**						
18–35	*ref*	*ref*	*ref*	*ref*	*ref*	*ref*
< 18	1.06	0.96	1.16	1.37	1.02	1.83
> 35	0.92	0.74	1.16	1.33	0.81	2.18
**Mother's height (centimeters)**						
< 145	*ref*	*ref*	*ref*	*ref*	*ref*	*ref*
145–149	0.74	0.67	0.82	1.08	0.81	1.43
> = 150	0.52	0.47	0.57	1.24	0.96	1.61
**Wealth quintile**						
Poorest	*ref*	*ref*	*ref*	*ref*	*ref*	*ref*
2	0.88	0.79	0.97	0.97	0.75	1.26
3	0.88	0.79	0.98	1.15	0.89	1.49
4	0.82	0.72	0.94	1.06	0.80	1.40
Least poor	0.78	0.70	0.88	1.09	0.82	1.46
**Interpregnancy interval (months)**						
18–36	*ref*	*ref*	*ref*	*ref*	*ref*	*ref*
< 18	1.16	1.06	1.26	1.05	0.86	1.29
> 36	0.92	0.81	1.05	0.82	0.63	1.07
No previous pregnancy	2.15	1.95	2.36	0.48	0.36	0.66
**Parity (still- and livebirths)**						
1–4	*ref*	*ref*	*ref*	*ref*	*ref*	*ref*
5 +	0.77	0.65	0.93	1.41	0.96	2.07
Prior pregnancy, parity 0	1.99	1.34	2.97	1.04	0.25	4.27
No previous pregnancy	NA			NA		
**Any Prior Livebirth Died**						
No prior livebirth death	*ref*	*ref*	*ref*	*ref*	*ref*	*ref*
Prior livebirth died	0.94	0.85	1.05	1.05	0.82	1.34
Prior pregnancy, no livebirth	0.94	0.63	1.38	0.48	0.15	1.55
No previous pregnancy	NA			NA		
**Number of antenatal care visits**						
No visits	*ref*	*ref*	*ref*	*ref*	*ref*	*ref*
1 visit	0.97	0.87	1.09	0.94	0.71	1.25
2–3 visit	0.92	0.83	1.01	0.84	0.67	1.05
4 or more	0.96	0.85	1.08	0.53	0.41	0.67
**Infant gender**						
Male	0.92	0.86	0.98	1.04	0.87	1.24
**Respiratory illness (ref = no)**						
2nd trimester	0.94	0.87	1.01	1.27	1.06	1.51
3rd trimester	1.04	0.94	1.14	0.67	0.54	0.84
**Poor appetite, vomiting (ref = no)**						
2nd trimester	1.24	1.16	1.32	0.84	0.69	1.00
3rd trimester	1.05	0.96	1.14	0.78	0.63	0.98
**Vaginal bleeding (ref = no)**						
2nd trimester	0.89	0.66	1.20	0.50	0.21	1.22
3rd trimester	1.30	0.88	1.91	0.68	0.09	4.86
**Swelling hands/face (ref = no)**						
2nd trimester	0.89	0.71	1.12	2.48	1.64	3.75
3rd trimester	0.83	0.71	0.97	1.11	0.75	1.66
**High Systolic (ref = no)**						
2nd trimester	1.11	0.72	1.70	0.38	0.08	1.79
3rd trimester	0.92	0.60	1.41	0.85	0.30	2.42
**High Diastolic (ref = no)**						
2nd trimester	0.89	0.66	1.19	1.64	0.93	2.87
3rd trimester	1.21	0.96	1.53	1.46	0.90	2.36
**Mean weight change in kilograms from 2**^**nd**^** to 3**^**rd**^** trimester**	0.93	0.92	0.95	0.92	0.88	0.96

*CI* Confidence interval, *LL* Lower limit, *UL* Upper limit, *ref* Reference category, *NA* Not applicable

Becoming pregnant 18 months or less time since the previous pregnancy was associated with SGA (OR: 1.16, 95% CI 1.07–1.27) as was having no previous pregnancy (OR: 2.12, 95% CI 1.93–2.34) compared to women with an IPI of 18–36 months. Having five or more births was protective (OR: 0.77, 95% CI 0.65–0.92) whereas having a previous pregnancy that did not result in a birth was associated with higher risk (OR 1.86, 95% CI 1.26–2.74). Having a prior live birth that later died was not associated with SGA.

Several of the pregnancy characteristics were associated with SGA status. Male babies had slightly lower odds compared to female babies (OR: 0.91, 95% CI 0.86–0.98). Every kilogram of weight gain between the second and third trimester was associated with a lower odds of SGA (OR: 0.93, 95% CI 0.92–0.95). Reported poor appetite/vomiting in the 2^nd^ trimester was associated with higher odds of SGA (OR: 1.27, 95% 1.19–1.35) Reporting swelling of the hands and face during the 3^rd^ trimester was negatively associated with SGA (OR: 0.81, 95% CI: 0.69–0.94). Other symptoms, vital signs and antenatal care seeking had no association with SGA.

### Risk factors for large-for-gestational age

In an inverse pattern to SGA, no previous pregnancy was protective for LGA (OR: 0.48 95% CI 0.35–0.66) and poor appetite/vomiting in the 3^rd^ trimester (OR: 0.78, 95% CI: 0.62–0.67) reduced the risk of LGA (Table 3).

Having four or more antenatal care visits protected against LGA (OR: 0.53 95% CI 0.41–0.68). Weight gain in the 2^nd^ and 3^rd^ trimesters was protective against LGA (OR: 0.92, 95% CI 0.87–0.96 for every kilogram gained). Reported respiratory illness in the 3^rd^ trimester was negatively associated with LGA (OR: 0.78, 95% CI: 0.62–0.97). Swelling (OR: 2.48, 95% CI: 1.64–3.75) in the 2^nd^ trimester and maternal age less than 18 years (OR: 1.39, 95% CI 1.03–1.87) were associated with LGA. None of the examined socio-economic factors were associated with LGA.

## Discussion

In this secondary analysis of a pregnancy cohort in rural Nepal, we found several statistically significant factors associated with the risk of SGA and LGA babies. We organized our findings using hypothesized causal models for SGA and LGA that groups the risk/protective factors by demographic/SES, obstetric and medical history, health status and health seeking behaviors, and index pregnancy characteristics.

### Demographic and socioeconomic status

We found caste associated with increased risk of SGA. Caste is a social construct, not a biological one, and this indicates presence of systemic factors such as discrimination impacting the heath of mothers and newborns, even after adjusting for SES and ANC visits. SGA was statistically significantly associated with several measures of poor socio-economic status such as fewer maternal years of school, poorer household wealth and caste, after adjusting for other biological and obstetric risk factors, a finding documented in both low-and high-income settings [25–28]. We found maternal stature greater than 145 cm to be protective of SGA, a finding also documented in LMICs [29]. Women with shorter stature have smaller pelvic size, which may restrict uterine growth. Also, shorter stature may be due to chronic malnutrition and is associated with poorer SES.

We did not find an association of SES or demographic characteristics with LGA. Poorer SES was found to be a risk factor for both SGA and LGA among poorer populations Brazil [30]. However, this could be due to differing stages in nutritional transition between Brazil and Nepal. The authors of the Brazil study noted obesity – a risk factor for LGA – was increasingly associated with poverty in these population, and this is not the case in rural Nepal [31].

### Obstetric and medical history

Interpregnancy intervals less than 18 months is a risk factor for SGA likely due to maternal depletion syndrome [32, 33]. No previous pregnancies and previous pregnancy with no live or stillbirth (miscarriage or abortion) are risk factors for SGA in this population. Nulliparity is a well-documented risk factor for SGA and this study shows women with gravidity still have higher risk of SGA if nulliparous [34]. We found grand multiparity (five or more births) not be a risk factor for SGA, similar to what was found in a meta-analysis of 41 studies – in fact it was found to be protective in this analysis [34]. We found no association with death of a prior livebirths and SGA. Nulliparity was found to be negatively associated with LGA (inverse finding of SGA).

### Index pregnancy characteristics & care seeking

Nausea and vomiting in the second trimester was a risk factor for SGA, also previously found in this population [35]. Poor appetite/vomiting was found to be “protective” for LGA. It is well documented that male gender fetuses and newborns are larger and heavier compared to female gender babies, a finding corroborated in our study as well [36]. We found no association with SGA between maternal age, hypertension, or symptoms of vaginal bleeding, respiratory infection and swelling reported during the pregnancy.

We did not find an association between antenatal care and SGA although it has been found to be protective in other settings [37]. We did find attending four or more visits of antenatal care to be protective for LGA. It is possible early detection and intervention of pregnancy-related hyperglycemia may be the under-laying cause. In 2014, over 90% of the pregnancy-related hyperglycemia occurred in low-income countries, and a quarter of all global cases are concentrated in South Asia [38]. A more recent study in Sri Lanka showed early detection of hyperglycemia in the first trimester, using the WHO diagnostic criteria, was associated with higher risk of LGA [39].

Our study found weight gain to be protective for both SGA and LGA. It is well established in the literature that gestational weight gain is associated with increased risk of LGA and protective for SGA [40, 41]. We estimated gestational weight gain by calculating the difference in average weights between the second and third trimesters. Women had an average of four visits during the second trimester and two visits during the third trimester. The timing of the visits during the trimester varied as well. It is possible that average weight by trimester was not stable and the variation in time between measurements may have reduced the precision and validity of this variable. We ran the model without the weight gain covariate and found similar results (Additional file 1. Appendix Table 1). Because the findings are in line with what is known about weight gain and SGA, we decided to leave this covariate in the model, but the association with LGA should be interpreted with caution.

Another unexplained finding was the association between respiratory infection and LGA. We decomposed the symptoms grouping and found it was third trimester cough that was “protective” of LGA and difficulty breathing in the second trimester was associated with increased LGA (data not shown). We did not find any evidence in the literature about respiratory infection symptoms and LGA. The association may be due to unmeasured confounding or the non-specific definition of respiratory infection (any reported coughing, difficulty breathing, wheezing or shortness of breath).

Another analysis on this same cohort found no additional neonatal mortality risk with LGA babies compared to appropriate-for-gestational age (adjusted hazard ratio: 0.76, 95% CIs: 0.56–1.03).^3^ We also found LGA to be less than 10 percent of the population and by definition, approximately a tenth of the babies are naturally large in a healthy population. Perhaps due to the low levels of maternal obesity or other factors, LGA status in this population does not indicate poor newborn/fetal health. This may also be why the associated risk/protective factors we found with LGA are not generalizable to other populations described in the literature.

### Limitations

There are important limitations in this study. This is a secondary data analysis; therefore, the original clinical trial was not developed to address this analysis. However, we have sufficient sample size to measure associations with both SGA and LGA with low levels of uncertainity. Gestational age was measured through LMP, not by the gold standard ultrasound in the first trimester, but LMP is considered adequate for determining gestational age in areas where ultrasound is not readily available [42, 43]. In the NOMS study, LMP dates were obtained early in pregnancy in most cases, reducing the likelihood of recall issues. Birthweight measures were not taken on very early neonatal deaths, more likely to be SGA. We addressed this by performing imputation of the birthweights, recalibrated to time at delivery, including all babies missing birthweight due to early neonatal death. The 95% confidence intervals take into account the uncertainity of performing the recalibration and imputation, so we consider this an appropriate analytical approach to address missing birthweights and birthweights measured post-delivery [19].

There may be measurement error for some of the covariates. For instance, symptoms are self-reported, retrospectively by the mother and there may be recall bias. We were also unable to include some important, documented risk factors for SGA and LGA such as pre-conception body mass index (BMI) and indoor air pollution [5, 44]. The lack of maternal weights early in pregnancy did not permit a complete calculation of weight gain or an analysis of how pre- or early-pregnancy BMI interacted with weight gain in regard to SGA or LGA. Antenatal care has been found protective of SGA and we found no association. It could be the quality of services is poor in this area so no impact on SGA was found.

## Conclusion

This is one of few population-based studies that evaluate risk factors of small- and large-for-gestational age in a low-income setting. We found high-risk pregnancies (nulligravida, gravida/nulliparous and short interpregnancy interval), nausea/vomiting symptoms in the second trimester, gestational weight gain, SES, fetal sex, and grand multiparity associated with SGA. Other associations of SGA/LGA with reported symptoms during pregnancy were difficult to interpret given the lack of biological plausibility and are likely due to measurement error. Four or more visits was protective of LGA and adolescent women had higher risk of LGA babies. Although LGA prevalence is low in this population, it may become a public health concern if maternal obesity increases.

Improving equitable access to high quality antenatal care throughout pregnancy will reduce prevalence of SGA babies. Women with high-risk pregnancies can be identified earlier for increased observation. Continuous monitoring of gestational weight gain and nausea/poor appetite symptoms throughout the pregnancy allows for earlier intervention. Nepal provides free maternal health services and has implemented a cash incentive program to encourage pregnant women to attend four or more visits of ANC since 2009 and similar programs can reduce socioeconomic inequities [45].

## Supplementary Information

**Additional file 1. **Appendix Table 1.

## Data Availability

The datasets analyzed during the current study available from the corresponding author on reasonable request.

## Abbreviations

AGA: Appropriate for gestational age
ANC: Antenatal care
BMI: Body mass index
CI: Confidence intervals
cm: Centimeters
IPI: Interpregnancy interval
IRB: Institutional review board
kg: Kilograms
LGA: Large for gestational age
LMICs: Low-and middle-income countries
LMP: Last menstrual period
mmHG: Millimeters of mercury
NOMS: Nepal oil massage study
OR: Odds ratio
pc: Percentile
SES: Socio-economic status
SGA: Small for gestational age

## Footnotes

1 Personal communication, Joanne Katz, Johns Hopkins Bloomberg School of Public Health, April 2022.

2 Personal Communication, Eric Ohuma, London School of Hygiene and Tropical Medicine, September 2021.

3 Personal communication, Tingting Yan, Johns Hopkins Bloomberg School of Public Health, April 2022.
